# Cases and Remarks on the External Application of Charcoal

**Published:** 1797

**Authors:** William Simmons

**Affiliations:** Member of the Corporation of Surgeons in London; and Surgeon to the Manchester Infirmary.


### VII. *Cases, and Remarks on the external Application of Charcoal; by Mr.* William Simmons, *Member of the Corporation of Surgeons of London, and Surgeon to the Manchester Infirmary.*

I WAS led to the use of charcoal powder in foul and fetid ulcers from perusing the second edition of Dr. Beddoes's publication on Factitious Airs, and am happy to add my testimony in its favour. From the untoward appearance of three stumps after amputation, which resisted the ordinary means of relief, I willingly gave it trial, and will now relate the result.

### CASE I.

W. Holt, aged 47, was admitted into the Infirmary April 27, for a carious ancle-joint, which was deemed proper for amputation. I accordingly removed it on Friday, the 1st of May, at the usual place above the ancle, and brought brought the integuments into contact so as to unite by the first intention.

An adhesion formed in the lower part of the wound in a few days, but elsewhere the appearance was unfavorable; the symptomatic fever ran high, and the parts covering the fibula ulcerated so as to expose the end of it. The common cataplasm was applied, and saline medicines, with opium, were given according to the indications. The disease, however, went on; the stump assuming a foul and gangrenous appearance, the discharge becoming very fetid, and the inflammation extending rapidly towards the knee. Bark, wine, and sulphuric acid were freely given; and opium, in such doses, and so repeated, as to ease pain; and the fermenting cataplasm was used instead of the common one. By vigorously pursuing these means for several days, the farther progress was checked, and healthy granulations began to appear; yet matter formed between the tibia and fibula in considerable quantity, and of a disagreeable smell. At this period, still continuing the bark and other remedies, the charcoal was substituted for the fermenting poultice. Two drachms of the powder being insufficient, half an ounce was added to enough of the common poultice, to cover the end of the ſtump, and as far as the inflammation extended. The diſcharge was henceforth not offenſive; but notwithſtanding the remedies were continued in ſuch doſes as his ſtomach would bear, his recovery was ſo ſlow as to render it expedient to ſend him into the country, where a ſpeedy amendment took place, and he was diſcharged in a few weeks with a good ſtump.

### CASE II.

Samuel Allwood, aged 23, was admitted at the ſame time for a caries in the left knee-joint, occaſioned by a blow, and of twelve years ſtanding. The degree of diſeaſe, and his exhauſted ſtate, ſanctioned the propriety of early amputation, which was accordingly performed on the ſame day as the former. At the end of ten days nearly a complete union had taken place, when unfavourable ſymptoms came on; the pulſe became frequent, with general languor, loſs of appetite, and a tendency to diſunion in the parts. The remedies given in the former caſe were exhibited in this, and the limb was wrapped up in the common poultice. Neverthelefs thelefs the recently-connected parts gave way, the whole put on a foul appearance, and produced a very fetid difcharge. The common being changed for the fermenting cataplafm, and the medicines continued, in a few days checked the progrefs of the fymptoms, yet the fmell of the matter was little mended. The charcoal was therefore applied as in the former cafe, and with equal benefit. Under this plan of treatment he was recovering, but fo flowly, that it was thought advifable to remove him into the country, where medicines were foon no longer neceffary, and in a few weeks he was difcharged with a tolerably good ftump.

The difpofition to ulceration in this cafe was fo ftrong, that a flannel roller, applied only fo tight as to prevent the retraction of the integuments, occafioned extenfive floughing, which fhewed no difpofition to exfoliate, although covered with the carbon, until the removal of the patient into purer air.

### CASE III.

James Nuttall, aged 17, was admitted on the fame day for a fcrophulous affection of the ancleancle-joint, which was adjudged, in confultation, proper to be removed. Accordingly I amputated the foot above the ancle on Friday, the 8th of May. In a week union, by the firft intention, had nearly taken place, and every thing promifed a fpeedy recovery; but fymptoms, fimilar to thofe of the former cafes, now came on, and increafed even to a greater degree of violence. The remedies ufed in the other cafes were given without the fmalleft benefit; and the pulfe became fo extremely rapid, that, together with the threatening appearance of the ftump, it left but flender hopes of a favourable iffue. Half an ounce of extract of bark was exhibited every twenty-four hours inftead of the powder, and the other means were perfevered in. The parts, however, difunited; and the integuments retracted, fo as to expofe the end of the tibia and fibula, were exquifitely tender to the touch, and difcharged a very fetid ichor. A trial was given to the common and fermenting poultices, and their ufe fuperfeded, as in the former cafes, by that of charcoal, which anfwered equally well in this inftance. The ulceration, however, continued to extend, and the irritation was extreme.

The patient was now removed into a more

 airy airy ward, by which, and a continuance of his medicines, the threatening ſymptoms ſomewhat abated till his removal into the country, where alſo a favourable change ſoon took place; but the integuments having retracted, ſo as wholly to uncover the end of the ſtump, and a variety of other untoward circumſtances occurring, he is not yet diſcharged. On that part of the ſound integument, covered by the poultice, a puſtulous eruption appeared, which was allowed to ſpread for ſeveral days, but being very ſore and painful, it was then dreſſed with the calamine cerate, which healed it in a few days.

Beſides uſing the remedies above mentioned, the patients were all allowed to eat freely of oranges.

For the introduction of fixed air into practice we are indebted to Dr. Macbride, to whom it was ſuggeſted by an obſervation of Sir John Pringle. It has ſince been employed in diſeaſes of a putreſcent tendency, in calculous caſes, and in foul and ill-conditioned ulcers. The carrot poultice, the fermenting cataplaſm, and ſtreams of fixed air, extricated from ſubſtances containing it, have been uſed in caſes where an ulcer has been foul, or the parts in a ſtate of gangrene. To apply the latter is oftentimes impracticable; the fermenting cataplafm, after lying on the part fome hours, becomes more offenfive than the fmell it was intended to cover; and carrots, which are not always to be met with, contain a refinous matter that may in fome inftances be improper. Charcoal, a fubftance containing carbonic acid gas, is free from thefe objections. In the cafes lately publifhed, it has been faid to poffefs the power of cleanfing and healing ulcers, as well as correcting the difcharge; the prefent cafes warrant no fuch conclufion. Holt and Allwood were better before it was ufed, and during the whole time powerful internal means were exhibited. The floughy ftate of Allwood's limb, covered by the carbon, led me to attend particularly to that circumftance, and to render that opinion at leaft doubtful. The cafe of Nuttall, however, is conclufive. Every means fuggefted to check the rapid progrefs of his diforder was ineffectual for fome time, and the ulceration extended, although the limb was covered with a thick poultice of the carbon. But what puts it beyond a doubt is the puftulous appearance which came upon and fpread on the found part of the limb covered by it, which was healed in a few days by the ceratum lapidis calaminaris.

 The

The change wrought by any ſubſtance on dead animal matter, is no proof of its operation on the living fibre. From the above caſes it appears that charcoal corrects the fetid ſmell of the matter iſſuing from a foul ulcer, but has no power in promoting cicatrization. The caſes related in the work above alluded to, are not quite ſatisfactory; for whilſt this ſubſtance was employed, remedies generally efficacious were adminiſtered, except in two inſtances which are ſo ſhortly reported as to prove nothing.

From its ſucceſs in foul ulcers I was induced to try it in ulcers attended with caries; and in theſe it has fully anſwered my expectations. It never fails to correct the ſtench, a circumſtance of great comfort to the patient himſelf and all around him. Beſides the unavoidable contamination of the air in apartments where many invalids are put together, the effluvia ariſing from foul and carious ulcers muſt have a pernicious effect, by adding to the impurity, and alſo tending to produce inbred diſeaſes. In ſome ulcers, where the diſcharge is not only fetid, but ſo acrimonious as to excoriate the neighbouring parts, I have found that it obtunds the acrimony; and as an ulcerated is the beſt abſorbing ſurface, may it not produce a farther farther benefit, by thus preventing a pernicious ſubſtance from being carried into the ſyſtem?

I am aware that three inſtances hardly corroborate evidence in favour of any remedy; but having ſince tried it in a great variety of caſes, I relate them as being the firſt, and which were very particularly attended to. Ample ſubſequent experience has convinced me that I have fairly appreciated its merits; to detail the many caſes in which I have tried it would be unprofitable labour, as their reſult correſponds with theſe.

It is hardly neceſſary to obſerve that the quantity of powder uſed ſhould be according to the extent of ſurface to be covered, and that it ſhould be well waſhed and dried to free it from ſalts or other foreign matter.

Changing the poultice once a day is ſufficient.

In the army and in military hoſpitals, where, from the number of ſurgical patients and other cauſes, attention to their caſes cannot be ſo regular, as in common practice, I apprehend it will be found a very valuable acquiſition.

*Mancheſter,*

*November* 10, 1795.

N. B. The Mancheſter Infirmary is ſituated in an airy and elevated part of the town; the

